# Diagnostic and Prognostic Role of Procalcitonin in Infections

**DOI:** 10.1100/tsw.2010.181

**Published:** 2010-10-01

**Authors:** Maria Hatzistilianou

**Affiliations:** School of Medicine, Aristotle University of Thessaloniki, Greece

**Keywords:** procalcitonin, pneumonia, neutropenia, bacterial infection, hematological malignancies, immunocompromised patients, cytokines

## Abstract

Despite several consensus conferences, the criteria for the definition of sepsis are still considered too sensitive and insufficiently specific. The traditional clinical signs of infection and routine laboratory tests used to diagnose bacterial infection and sepsis lack diagnostic accuracy and can be misleading, particularly in patients with immunodeficiencies. The problems with sepsis definitions and diagnoses are indications of the need to focus on biochemical mediators capable not only of distinguishing the inflammatory response to infection from other types of inflammation, but also of indicating the severity and prognosis of the disease. Thus, physicians need an early and rapid marker for detecting bacterial infection and distinguishing it from viral infection. Several studies revealed that elevated procalcitonin (PCT) levels in human blood could be detected in cases of sepsis and bacterial infection. PCT is a protein that can act as a hormone and a cytokine. It can be produced by several cell types and many organs in response to proinflammatory stimuli, particularly bacterial infection. It provides a rapid diagnostic test, available at the patient's bedside, and its half-life is suitable for daily monitoring of the disease progress.

## INTRODUCTION

Procalcitonin (PCT) is a 116-amino-acid residue peptide with molecular weight of about 13 kDa[1]. The amino acid sequence of PCT was first described by Le Moullec et al. in 1984[1]. In 1993, PCT was described as a new and innovative parameter of infection[2]. However, an initial publication in 1983 first called attention to increased serum levels of immunoreactive PCT in patients with the staphylococcal toxic shock syndrome, a severe form of sepsis[3].

Production is governed by the calcitonin I (CALC-I) gene on chromosome 11p15.2-p15.1. It is a gene with six exons, although the first exon is not apparently translated[4,5].

In the traditional endocrine view, PCT is produced mainly in neuroendocrine C cells of the thyroid. In the absence of infection, the extrathyroidal transcription of the CALC-I gene is suppressed and confined to selective expression in neuroendocrine cells found mainly in the thyroid and lung. In these neuroendocrine cells, the mature hormone is processed and stored in secretory granules[4,5]. A microbial infection induces a ubiquitous increase in CALC-I gene expression and a constitutive release of PCT from all extrathyroidal tissues and cell types throughout the body, including liver, kidney, pancreas, and adipose and white blood cells[6].

The mechanism proposed for PCT production after inflammation and its role are still not completely known[7]. During microbial infection, there is an increase of CALC-I gene expression that causes release of PCT from all parenchymal tissues and from differentiated cell types throughout the body. The inflammatory release of PCT can be induced in two main ways: one (direct way) is due to toxins or lipopolysaccharides released by microbes and the other (indirect way) is through cell-mediated host response mediated by inflammatory cytokines (e.g., interleukin-1b [IL-1b], interleukin-6 [IL-6], tumor necrosis factor-α [TNF-α])[8,9,10].

It is of interest that, physiologically, newborns also exhibit a considerable increase in circulating PCT that reverses spontaneously during the first week[11,12,13]. This could be interpreted as a host response to the initial establishment of the normal intestinal bacterial flora.

Plasma PCT was shown to be very low in healthy individuals (<0.5 ng/ml), while it elevates in severe bacterial infections and septic conditions, where it can reach up to 1,000 ng/ml without changes in serum calcitonin levels[14,15,16]. It is also reported to correlate with the severity and clinical outcome of infection[17]. However, increased PCT levels may not always be related to systemic infection[18].

Noninfectious conditions that may increase PCT levels include (a) major trauma, surgical trauma (including extracorporeal circulation), and burns; (b) the first 2 days of a neonate's life; (c) medullary C-cell carcinoma, small cell lung carcinoma, or bronchial carcinoid; (d) treatment with OKT3 antibodies, interleukins, TNF-α, and other drugs stimulating the release of proinflammatory cytokines; and (e) prolonged or severe cardiogenic shock, prolonged severe perfusion anomalies, Child-Pugh Class C liver cirrhosis, and peritoneal dialysis treatment.

This article is intended to address certain issues related to PCT and to discuss the role of PCT in the diagnosis of infections, in the differential diagnosis among various infection types, and in monitoring and prognosis of bacterial infections.

## DIAGNOSTIC ROLE OF PCT

Fever is one of the most common reasons for visits to an emergency department, particularly for children younger than 3 years of age. Children of this age with febrile illnesses present severe bacterial infections (SBIs) 10–25% and the diagnosis may be confusing because clinical findings often provide inadequate information, especially if localizing findings are absent[19]. Therefore, there is a need for sensitive and specific laboratory markers of infection. Andreola et al. found that PCT, but not C-reactive protein (CRP), concentrations were significantly higher in the group of children with more invasive bacterial infections, e.g., sepsis and meningitis, suggesting that PCT is not just a marker of infection, but, more importantly, an appropriate marker of the severity of infection[20]. They also found that in patients with fever evolution <8 h, PCT may perform better than CRP ($p < 0.05$). Moreover, they found that PCT levels rise more rapidly than CRP levels. These findings are in accordance with results obtained in other clinical studies[21,22,23,24].

Another field in which PCT plays a significant diagnostic role is childhood pneumonia[25], which remains a common and serious illness among children. Its etiological diagnosis is optimal for the determination of the initial treatment. The pathogen is not defined in the majority of patients with pneumonia and the treatment is empirical[25,26]. An increased number of antimicrobial drugs have been given for a long time, with doubtful results. On the other hand, the widespread use of antibiotics for nonbacterial infections has led to antibiotic resistance. In order to reduce this phenomenon, antibiotic use must be limited to infections of bacterial etiology[25,26,27]. However, identifying clinically the etiology of pneumonia in children is difficult because single clinical, radiological, or laboratory parameters have a limited value in predicting the infectious organism[25,28]. Thus, clinicians need early markers that are capable of reflecting the severity of infection and distinguishing bacterial vs. viral pneumonia.

In one of our studies involving children with bacterial and viral pneumonia, we found that, on admission, 100% of the children with bacterial pneumonia had elevated values of PCT (0.94–62.10 ng/ml), while only 34% of the children with viral pneumonia had PCT levels between 0.5–2.13 ng/ml[25]. Also, we pointed out that a PCT concentration of more than 2 ng/ml had 100% sensitivity, 98% specificity, and 93% positive predictive value for children with bacterial pneumonia and may help to distinguish bacterial from viral pneumonia[25]. In contrast, the levels of CRP in bacterial pneumonia were elevated in all patients (100%) and in viral pneumonia (88% of patients), CRP had a high sensitivity 96%, low specificity 38%, and low predictive positive value 42%[25]. The above results indicate that PCT is a good marker not only for screening the infectious diseases, but also for distinguishing between bacterial and viral infections. These results are in accordance with those of other studies[25,26,27,28].

Moreover, PCT plays a significant role in the diagnosis of infections in immunosuppressed patients[29,30,31,32,33,34]. Neutropenia as a state of immunosuppression is probably the major problem in children with hematological malignancies undergoing intensive chemotherapy. During neutropenia, the morbidity and mortality rates due to bacterial infections are high. Thus, the early diagnosis of bacterial infections must be accurate. However, the early diagnosis is very difficult because the typical clinical features of bacterial infections, and routine laboratory tests used to diagnose bacterial infection and sepsis (e.g., CRP, white blood cell, or lactate), lack diagnostic accuracy and can be misleading[30,31,32, 33,34,35,36,37]. Also, neutropenic children with infection may or may not have fever. However, in these patients, fever may be due to other causes than infections, such as the underlying disorder, or the administration of drugs or blood products[30,31,32,33]. The early diagnosis of febrile neutropenic patients at high risk for severe infections would be helpful in management decisions regarding antimicrobial therapy and hospitalization[30,33]. Thus, clinicians need early markers that are capable of reflecting the severity of infection and distinguishing episodes of high and low risk for septic complications[31,35,36,37].

In our studies that included children with hematological malignancies undergoing intensive chemotherapy, we point out that, based on a cutoff level of 2 ng/ml PCT, groups with bacterial infections showed a sensitivity and specificity of 94 and 96.5%, respectively, for predicting bacteremia. In comparison with other markers, such as CRP, IL-6, IL-8, IL-1b, sTNFRII, TNF-α, and soluble adhesion molecules, PCT was a better marker, in any case, taking into account its specificity and sensitivity[30,31,32,33,34]. The discriminatory power of PCT and the above markers in neutropenic patients was evaluated in terms of the area under the receiver operating characteristic (ROC) curve (AUC) (AUC for PCT = 0.875, CRP = 0.708, IL-6 = 0.895, IL-8 = 0.750, IL-1b = 0.616, sTNFRII = 0.795, TNF-α = 0.777, sICAM-1 = 0.579, sVCAM-1 = 0.598, sE-selectin = 0.505, sP-selectin = 0.579). PCT was the best discriminator for bacterial infections[30,31,32,33,34].

Another reason that PCT is a valuable marker of bacterial infection is the fact that PCT may be determined easily and quickly by any doctor at the patient's bedside and without any special equipment except a simple centrifuge. This determination is done by the semi-quantitative method called PCT-Q at the onset of infection. If PCT-Q is positive, it must be continued by serial quantitative determination of PCT levels by classical methods[38,39].

In another study, we compared the two methods for determination of PCT in the serum of patients with infection and we found high correlation between these two methods[38]. Measuring PCT-Q levels in patients made it possible to reduce the number and the duration of hospitalizations, to reduce excessive antibacterial treatment, and, finally, to reduce the bacterial resistance to antibacterial treatment.

## PROGNOSTIC ROLE OF PCT

It has been shown that the level of PCT in serum increases significantly during an infection of bacterial origin[2]. Today, PCT is considered to be one of the earliest and most specific markers of bacterial infections. However, PCT is not only a specific marker of infection, but is also useful in monitoring the host response to the infection and to the treatment[26,28,30,31,32,33,34,40]. Thus, PCT levels must be determined serially. If PCT levels decrease by more than 30% of the initial value after the first 24 h from the onset of antibacterial treatment, it indicates that the treatment is the appropriate one and the infection is under control[32,33]. If PCT levels increase, it means that the antimicrobial treatment must be changed. If PCT levels continuously increase, the host response to infection is very poor and the host immune system must be reinforced[32,33].

PCT can reduce the days of antibacterial treatment. In a randomized trial to determine the length of antibiotic therapy using a laboratory parameter carried out in 2006[26], Christ-Crain et al. point out that using PCT as therapy guidance can actually reduce the length of therapy from 12 to 5 days, and the duration of antibiotic therapy was shortened by 65% with a similar outcome in patients independent of the severity of community-acquired pneumonia.

A randomized, open, multicenter trial carried out from December 2004 to April 2006 showed that PCT can also be used to decrease antibiotic therapy outside the hospital setting[28]. The reduction in duration of therapy applied to patients in all pneumonia severity groups. Outcomes were similar for both groups, with no difference in duration of hospitalization, quality-of-life score, intensive care unit admission, or pneumonia-related mortality.

A study conducted by Franzin and Cabodi showed that PCT is increased in Legionella pneumonia and, despite its nonspecificity, it can be used as a prognostic marker[41]. These results are in concordance with those of a study carried out by Haeuptle et al.[42].

Changes in biomarker levels during the course of the disease may also assist physicians to identify those patients at risk of deterioration and progression to severe bacterial infection[28,41]. The authors point out that nonsurviving patients had significantly higher PCT levels on day 1 than surviving patients and they found that with serial PCT measurement, surviving patients had a decrease in PCT levels by day 3, whereas nonsurvivors had an increase.

## CONCLUSIONS

Identifying patients with bacterial infection and sepsis is a major challenge in emergency departments and critical care units, where mortality from sepsis remains high due to delayed diagnosis and treatment. The physician needs a biological marker for early diagnosis of the bacterial infections. Today, PCT is considered to be one of the earliest and most specific markers of sepsis in immunocompromised and immunodeficient patients. It provides a rapid diagnostic test, available at the patient's bedside, and its half-life is suitable for daily monitoring of disease progress. However, PCT is not a substitute for a careful history and physical examination, and alone is not an absolute criterion for deciding about the hospitalization and administration of antibacterial treatment.
